# 6-Shogaol Inhibits Oxidative Stress-Induced Rat Vascular Smooth Muscle Cell Apoptosis by Regulating OXR1-p53 Axis

**DOI:** 10.3389/fmolb.2022.808162

**Published:** 2022-01-31

**Authors:** Jing Liu, Bin Li, Wenlian Li, Taowen Pan, Yunpeng Diao, Fangjun Wang

**Affiliations:** ^1^ College of Pharmacy, College of Integrative Medicine, Dalian Medical University, Dalian, China; ^2^ Key Laboratory of Separation Sciences for Analytical Chemistry, Dalian Institute of Chemical Physics, Chinese Academy of Sciences, Dalian, China; ^3^ Dalian Anti-Infective Traditional Chinese Medicine Development Engineering Technology Research Center, Dalian, China

**Keywords:** vascular smooth muscle cell, OXR1, p53, oxidative stress, apoptosis, 6-shogaol (PubChem CID: 5281794)

## Abstract

Apoptosis of vascular smooth muscle cells (VSMCs) is closely related to the pathogenesis of cardiovascular diseases, and oxidative stress is an important cause of VSMCs’ death. Inhibiting VSMCs apoptosis is an effective preventive strategy in slowing down the development of cardiovascular disease, especially for atherosclerosis. In this study, we found that oxidation resistance protein 1 (OXR1), a crucial participator for responding to oxidative stress, could modulate the expression of p53, the key regulator of cell apoptosis. Our results revealed that oxidative stress promoted VSMCs apoptosis by overexpression of the OXR1-p53 axis, and 6-shogaol (6S), a major biologically active compound in ginger, could effectively attenuate cell death by preventing the upregulated expression of the OXR1-p53 axis. Quantitative proteomics analysis revealed that the degradation of p53 mediated by OXR1 might be related to the enhanced assembly of SCF ubiquitin ligase complexes, which is reported to closely relate to the modification of ubiquitination or neddylation and subsequent degradation of p53.

## Introduction

Nowadays, cardiovascular disease remains the major cause of morbidity and mortality worldwide (Townsend et al., 2016). Increasing evidence had suggested the contribution of the vascular smooth muscle cells (VSMCs) apoptosis to the pathogenesis of cardiovascular diseases, including hypertension, atherosclerosis, and even myocardial infarction (Kumar et al., 2014; Mill et al., 2014). Therefore, inhibiting VSMCs apoptosis could be a potential preventive strategy in slowing down the development of cardiovascular disease, especially for atherosclerosis (Yan et al., 2017). As oxidative stress is considered one of the most important pro-apoptotic stimuli leading to VSMCs apoptosis in atherosclerotic plaques (Korshunov and Berk, 2008), an effective antioxidant is imperatively needed to attenuate the oxidative stress-induced VSMCs apoptosis.

Oxidation resistance protein 1 (OXR1), a crucial participator for the early response to oxidative stress (Natoli et al., 2008), has been considered an important antioxidant protein that regulates the expression of a variety of antioxidant enzymes and can prevent oxidative damage caused by various oxidative stresses in different tissues (Li et al., 2014; Svistunova et al., 2019). However, there is no evidence about its physiological function in VSMCs. Recently, OXR1 has been reported to modulate apoptosis and cell cycle upon hydrogen peroxide (H_2_O_2_) exposure (Yang et al., 2015), but the regulatory mechanism is still unclear as well.

Apoptosis is an evolutionarily conserved physiological form of cell death, which is precisely controlled by a genetic program *via* the activation and inactivation of specific genes (Bennett et al., 1995). The tumor suppressor protein p53 is one of the key regulators of apoptosis in a wide variety of cell types and organisms. In response to exogenous cellular stress, p53 is activated to regulate the expression of numerous genes involved in cellular outcomes, including cell cycle arrest and cell death (Laptenko and Prives, 2006). A dual role has been demonstrated for p53 in VSMCs. The overexpression or activation of p53 induced by apoptotic stimuli could mediate VSMCs apoptosis while the endogenous level of p53 protects VSMCs from apoptosis (Mercer et al., 2005).

6-Shogaol (6S) is a major biologically active compound present in ginger (the rhizome of *Zingiber officinale*) and exhibits important pharmacological effects, including anti-oxidative, anti-inflammatory, neuroprotective, and anti-apoptosis (Kim and Kwon, 2013; Kim et al., 2014). It is reported that 6S could attenuate the apoptosis of neurons and hepatocytes induced by oxidative stress (Kim and Kwon, 2013; Kim et al., 2014) and prevent cardiovascular disease by protecting endothelial cells against oxidized LDL-induced injuries (Wang et al., 2013). In the present study, 6S exhibited strong anti-apoptotic activity in VSMCs and has an inhibitory effect on the overexpression of OXR1 induced by H_2_O_2_. The results of quantitative phosphoproteomic analysis and western-blot revealed the intimate relationship between the anti-apoptotic activity of 6S and the inhibition of overexpression/activation of p53, indicating a compact relationship between OXR1 and p53. Furthermore, the modulation of p53 expression in VSMCs regulated by OXR1 was confirmed, and the quantitative proteomic analysis suggested that OXR1 might adjust p53 degradation through assembling SCF ubiquitin ligase complexes. Overall, our results showed that 6S protected VSMCs against oxidative stress-induced apoptosis by regulating the OXR1-p53 axis and 6S represented a promising compound for preventing atherosclerosis.

## Methods

### Cell Culture

The Rat arterial vascular smooth muscle cell (VSMC) was obtained from the Cell Bank of Chinese Academy of Sciences (Shanghai, China) and cultured in Dulbecco’s modified Eagle medium (DMEM), including 4.5% glucose (Gibco, Grand Island, United States) and supplemented with 10% fetal bovine serum (FBS, Gibco, Grand Island, United States), 100 IU/ml penicillin and 100 μg/ml streptomycin at 37°C with 5% CO_2_. 6S (Aladdin, Shanghai, China) was dissolved in DMSO with a high concentration of 100 mM and stored at −80°C as a stock solution. Hydrogen peroxide (H_2_O_2_, Solarbio, Beijing, China) and 6S was diluted freshly in a growth medium immediately before use. Cells were treated with H_2_O_2_ or 6S following recovery in fresh medium for the indicated time.

### Cell Viability Assay

Cell Counting Kit-8 (CCK-8, Beyotime, Shanghai, China) was employed to determine the cell viability of VSMCs. VSMCs were seeded in 96-well plates and grown to an 80% confluence in complete medium. Then, VSMCs were performed with a series of treatments, replaced with fresh medium containing 10% CCK-8 solution, and incubated for 3 h at 37°C with 5% CO_2_. The optical density (OD) of each well was determined under 450 nm wavelength with the Varioskan Flash (Thermo Scientific, United States).

### Determination of SOD Activity, MDA Content, and LDH Leakage in H_2_O_2_-Induced VSMCs

The VSMCs were exposed to 1 mM H_2_O_2_ for 2 h after incubation with 6S (0, 0.5, 2, 8 μM) for 24 h. Then, the SOD activity, MDA content, and LDH leakage were measured by commercial chemical assay kits (Nanjing Jiancheng Bioengineering Institute, Nanjing, China) according to the manufacturer’s instructions.

### siRNA-Mediated OXR1 Knockdown

The OXR1 siRNA was synthesized by GenePharma (Suzhou, China) and transfected the VSMCs using siRNA-mate-plus following the manufacturer’s recommendations. The sense sequence for the OXR1 siRNA was 5′-GGA​GAU​AAA​GCU​ACA​GGA​ATT-3′, and the antisense sequence was 5′- UUC​CUG​UAG​CUU​UAU​CUC​CTT-3′. The sense sequence of the negative-control siRNA was 5′-UUC​UCC​GAA​CGU​GUC​ACG​UTT-3′, and the antisense sequence was 5′-ACG​UGA​CAC​GUU​CGG​AGA​ATT-3′. After 24 h transfection, the cells were exposed to 1 mM H_2_O_2_ for 2 h after pre-treated with different concentrations of 6S for 24 h.

### Western Blot Analysis

The RIPA lysis buffer (Beyotime, Shanghai, China) was used to lyse the cell pellets and then collected the supernatants and determined the protein concentrations by BCA protein assay kit (Beyotime, Shanghai, China). The protein extracts were separated by SDS-PAGE, transferred to a polyvinylidene fluoride (PVDF) membrane (Millipore, MA, United States), and incubated with specific antibodies of OXR1 (Proteintech, 13514-1-AP), p53 (Proteintech, 10442-1-AP), p21 (Proteintech, 28248-1-AP), Bax (Proteintech, 50599-2-Ig), cleaved caspase-3 (Proteintech, 19677-1-AP), and GAPDH (Proteintech, 10494-1-AP) at dilution of 1:500 incubated at 4°C overnight after being blocked with 5% non-fat dry milk in Tris-buffered saline containing 1% Tween-20 (TBST) at room temperature for 2 h. Then, the membranes were incubated with secondary antibody at room temperature for 2 h and exposed to the enhanced chemiluminescence-plus reagents according to the manufacturer’s protocol after extensive washing. Finally, the protein bands were detected using Bio-Rad Image Studio and normalized against GAPDH.

### Statistical Analysis

All the data were presented as the mean ± standard deviation (SD) and analyzed by one-way analysis of variance with Tukey’s multiple comparison tests in GraphPad Prism 5.0 software. *p* < 0.05 was considered statistically significant.

### Digestion, Stable Isotope Dimethyl Labeling, and Phosphopeptides Enrichment

The protein extracts of control, H_2_O_2_ injury, and 6S treatment (8 μM) groups were precipitated with five volumes of ice-cold acetone/ethanol/acetic acid (v/v/v = 50/50/0.1) at −20°C overnight. The precipitated protein was collected by centrifuging at 15,000 g for 30 min and washing with acetone and 75% ethanol in turn. Then, the protein sample was re-dissolved with 8 M urea/100 mM TEAB (pH 8.0) solution, and the protein concentration was determined by BCA assay. After reduction by 10 mM DTT at 60°C for 1 h and alkylation by 20 mM IAA in the darkness at room temperature for 30 min, the protein was eight times diluted with 100 mM TEAB buffer and digested by trypsin (enzyme/substrate, 1/25 (w/w)) overnight at 37°C.

Stable isotope dimethyl labeling and phosphopeptides enrichment were performed in solution according to Boersema et al. (2009) and Zhou et al. (2013). Equal amounts of protein digests from control, H_2_O_2_ injury, and 6S treatment (8 μM) groups were labeled with light (L), medium (M), and heavy (H) stable isotope dimethyl labels, and the equal amounts of protein digests from control and siRNA group were labeled with light (L) and medium (M) stable isotope dimethyl labels. The labeling reactions were performed by adding light labeling reagents (5 mM CH_2_O and 5 mM NaCNBH_3_), medium labeling reagents (5 mM CD_2_O and 5 mM NaCNBH_3_), and heavy labeling reagents (5 mM ^13^CD_2_O and 5 mM NaCNBD_3_) at 25°C for 1 h, respectively. 10% (v/v) ammonia solution was added to quench the labeling reactions, and then 10% (v/v) formic acid was added to further quench the reactions and acidify the samples. Then, the labeled samples were mixed for further usage. For phosphopeptides enrichment, the sample was mixed with equal volume of 80% ACN/6% TFA solution containing Ti^4+^-IMAC microspheres. After shaking for 30 min, Ti^4+^-IMAC microspheres were collected by centrifugation and rinsed with 50% ACN/6% TFA/200 mM NaCl and 30% ACN/0.1% TFA, respectively. Finally, the phosphopeptides were eluted with 10% (v/v) ammonia solution and lyophilized for analysis by LC-MS/MS.

### LC-MS/MS Analysis and Database Searching

The samples were analyzed by LTQ Orbitrap Velos (Thermo, San Jose, CA, United States) with Accela 600 HPLC system (Thermo, San Jose, CA, United States) as described previously (Liu et al., 2017) with minor modifications. The samples were loaded on a C18 capillary trap column (200 μm i.d. × 4 cm) packed with C18 AQ beads (5 μm, 120 Å). 15 cm × 75 µm i.d. analytical column packed with C18 AQ beads (3 μm, 120 Å) was used for LC separation. The buffers used for online separation were 0.1% (v/v) formic acid in water and 0.1% (v/v) formic acid in ACN. The flow rate was 200 nl/min for the nanoflow LC-MS/MS analysis. The gradient from 5 to 35% (v/v) ACN was performed in 150 min. The MS and MS/MS spectra were collected by CID at 35% energy in a data-dependent mode of 1 MS scan from 400 to 2,000 m/z with the mass resolution of 60,000 at m/z 400 in the Orbitrap analyzer followed by 20 MS/MS scans in the ion trap. The dynamic exclusion repeat count was 1 with duration time of 30 s, the exclusion list size was 500 with exclusion time of 90 s, and the charge state rejection function was enabled to reject the ions with single and “unassigned” charge states.

The obtained raw files were searched using MaxQuant (version 1.3.0.5) (Cox and Mann, 2008) with a reviewed UniProt protein FASTS database of Rat (*Rattus norvegicus, 8,133 sequences*). Cysteine carboxamidomethylation was set as a static modification, and methionine oxidation, acetylation of protein N-term, and phosphorylation (STY) were set as variable modifications with up to two missing cleavages of trypsin allowed. Precursor ion mass tolerances were 20 ppm, and fragment ion mass tolerance was 0.8 Da. The false discovery rates (FDR) for peptide, protein, and protein phosphorylation sites were all set as < 1%, and a minimum length of six amino acids was used for peptides identification. Triplets were selected as the quantification mode with the dimethyl Lys 0 and N-term 0 as light labels, dimethyl Lys 4 and N-term 4 as medium labels, and dimethyl Lys 8 and N-term 8 as heavy labels. All other parameters are the default setting in MaxQuant. After filtering by the FDR ≤ 1%, only phosphorylation sites with location probability ≥0.75 and score difference ≥5 were considered as highly reliable results and used for further investigation. Finally, the results were imported into the MaxQuant associated software suite Perseus (Tyanova et al., 2016) for further analysis.

### ProteomeXchange Accession

The mass spectrometry proteomics data were deposited to the ProteomeXchange Consortium via the PRIDE partner repository with the dataset identifier PXD015127.

## Results

### 6S Significantly Attenuated Oxidative Stress-Induced Apoptosis of VSMCs

Oxidative stress is one of the most important stimuli that induce cardiovascular disease as ROS can act as second messengers in pathways leading to cellular apoptosis, DNA synthesis, and proto-oncogene mRNA expression in VSMCs (Irani, 2000; Cao et al., 2015). Nowadays, H_2_O_2_ is widely used as a rapid and sensitive oxidative stress-induced apoptotic model (Zeng et al., 2018) because it is stable and easily penetrates the cell membrane. In this study, the H_2_O_2_ model was carefully examined and then used to simulate oxidative stress state in VSMCs. There was a significant dose-dependent decrease in cell viability after exposure to different concentrations of H_2_O_2_ for 2 h. When cells were incubated with 0.8 and 1 mM H_2_O_2_, cell viabilities started to level off and attenuated more than 50% when compared with the control group (Supplementary Figure S1). Hence, 1 mM H_2_O_2_ was selected as the appropriate concentration for inducing oxidative stress and apoptosis in VSMCs in the subsequent experiments.

**FIGURE 1:**
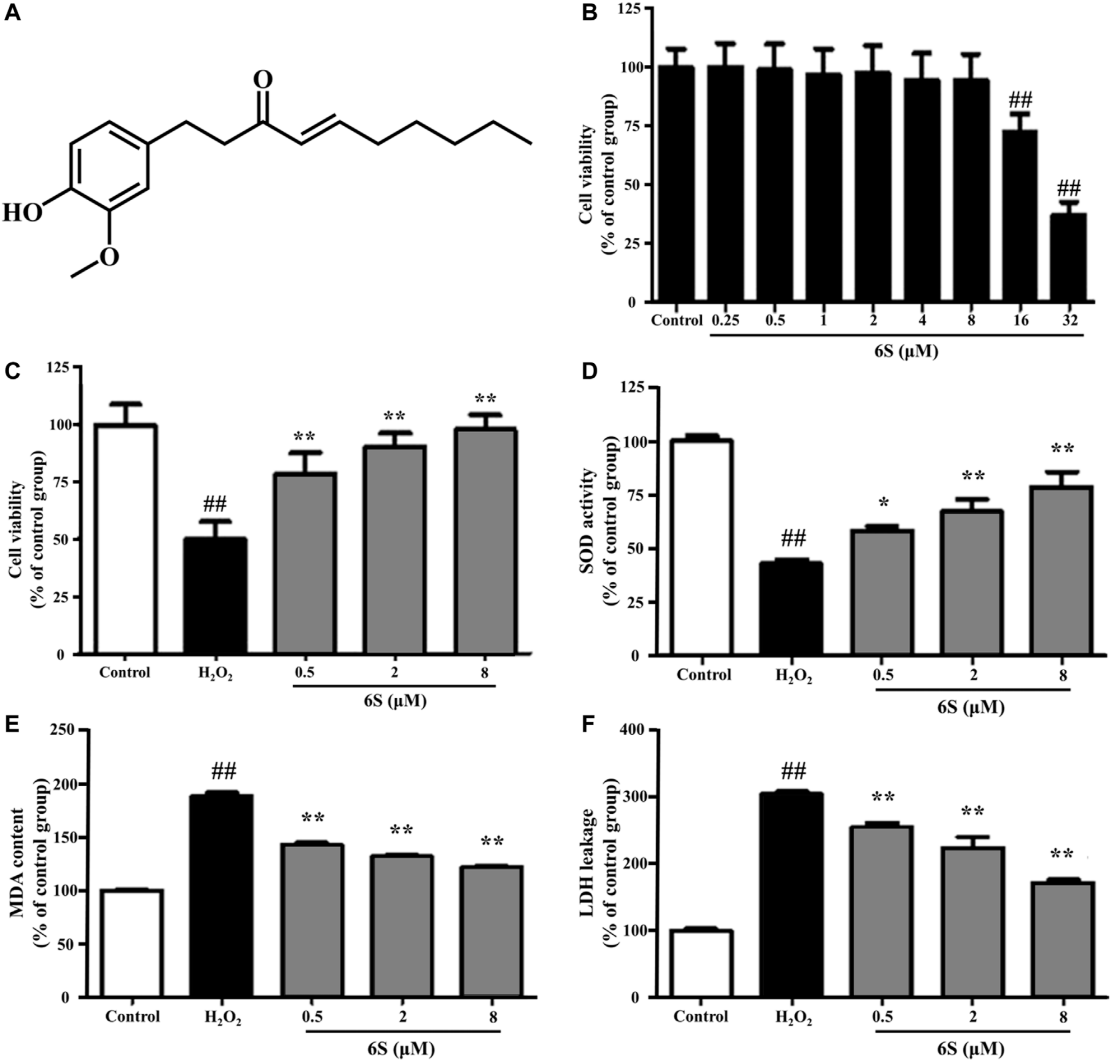
The effect of 6S on cell viability, SOD activity, MDA content, and LDH leakage in VSMCs. **(A)** Chemical structure of 6S. **(B)** The cell viability of VSMCs after incubation with 0, 0.25, 0.5, 1, 2, 4, 8, 16, or 32 μM 6S for 24 h. **(C)** The cell viability, **(D)** SOD activity, **(E)** MDA content, and **(F)** LDH leakage of VSMCs incubated with 0, 0.5, 2, and 8 μM 6S for 24 h followed with exposure to 1 mM H_2_O_2_ for 2 h. All the data were expressed as mean ± SD (*n* ≥ 3). ##*p* < 0.01 versus control group. **p < 0.01 versus H_2_O_2_ group.

We examined the cytotoxic effect of 6S, and the results showed that 6S had a little cytotoxic effect on VSMCs until its concentration was higher than 16 μM (Figure 1B). We next examined the protective effect of 6S on H_2_O_2_-induced cell injury, and the results revealed that 6S clearly decreased H_2_O_2_-induced cell injury in a concentration-dependent manner (Figure 1C).

**FIGURE 2:**
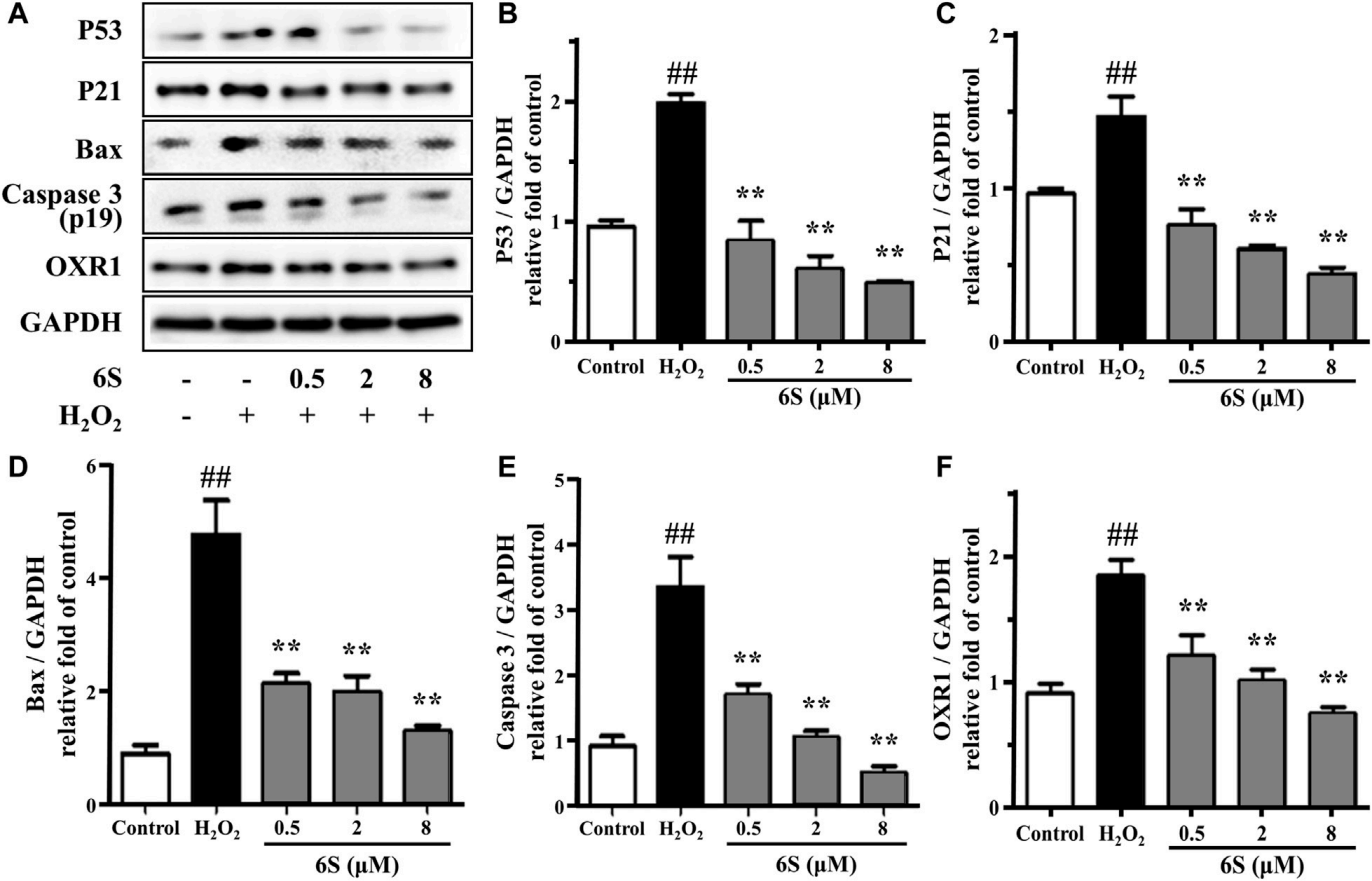
The effect of 6S on protein expressions of p53, p21, BAX, cleaved caspase-3 (p19), and OXR1. VSMCs were incubated with 0, 0.5, 2, and 8 μM 6S for 24 h followed by exposure to 1 mM H_2_O_2_ for 2 h. All the data were expressed as mean ± SD (*n* = 3). ##*p* < 0.01 versus control group. **p < 0.01 versus H_2_O_2_ group.

To investigate the potential anti-oxidative effect of 6S on VSMCs, the SOD activity, MDA content, and LDH release from VSMCs were determined. As shown in Figures 1D–F, H_2_O_2_ significantly decreased SOD activity, which is an important enzyme for preventing oxidative damage, but increased MDA levels, the metabolic end-product of lipid peroxides which could reflect the degree of the oxidation reaction and induce cell damage, and LDH leakage, a representation of cell death, in VSMCs. Encouragingly, the above indexes were markedly alleviated by the pre-treatment with 6S in a dose-dependent manner (Figures 1D–F), indicating that 6S could significantly attenuate the H_2_O_2_-induced oxidative stress. Further, we detected the expression levels of key proteins involved in the process of cell apoptosis. The expression levels of BAX and cleaved caspase-3 (p19) were significantly increased upon H_2_O_2_ treatment, indicating that H_2_O_2_ successfully induced VSMCs apoptosis. Fortunately, their expression levels were attenuated by pre-treatment with 6S in a dose-dependent manner (Figure 2), indicating the decreased apoptosis rate. Above all, these results suggested that 6S had a strong protective effect on H_2_O_2_-induced cell apoptosis.

### 6S Inhibited the Expression of OXR1 in Rat VSMCs Exposed to H_2_O_2_

OXR1 has been considered a crucial participator for responding to oxidative stress. It participates in the detoxification of ROS and exhibits an important role in protecting various human cells against oxidative damage (Li et al., 2014; Svistunova et al., 2019). Different from previous reports that OXR1 expression was suppressed under stress (Ingram et al., 2007; Yang et al., 2018), OXR1 showed upregulation upon H_2_O_2_ exposure in the present study. Then, pre-treatment with 6S effectively reversed the upregulation of OXR1 in a dose-dependent manner (Figure 2), indicating that OXR1 might play important roles in the protective effects of 6S against apoptosis. However, the relative mechanism was still unknown.

### Mass Spectrometry-Based Quantitative Phosphoproteomic Profiling of Oxidative Stress-Induced Apoptosis and 6S Protection

**FIGURE 3:**
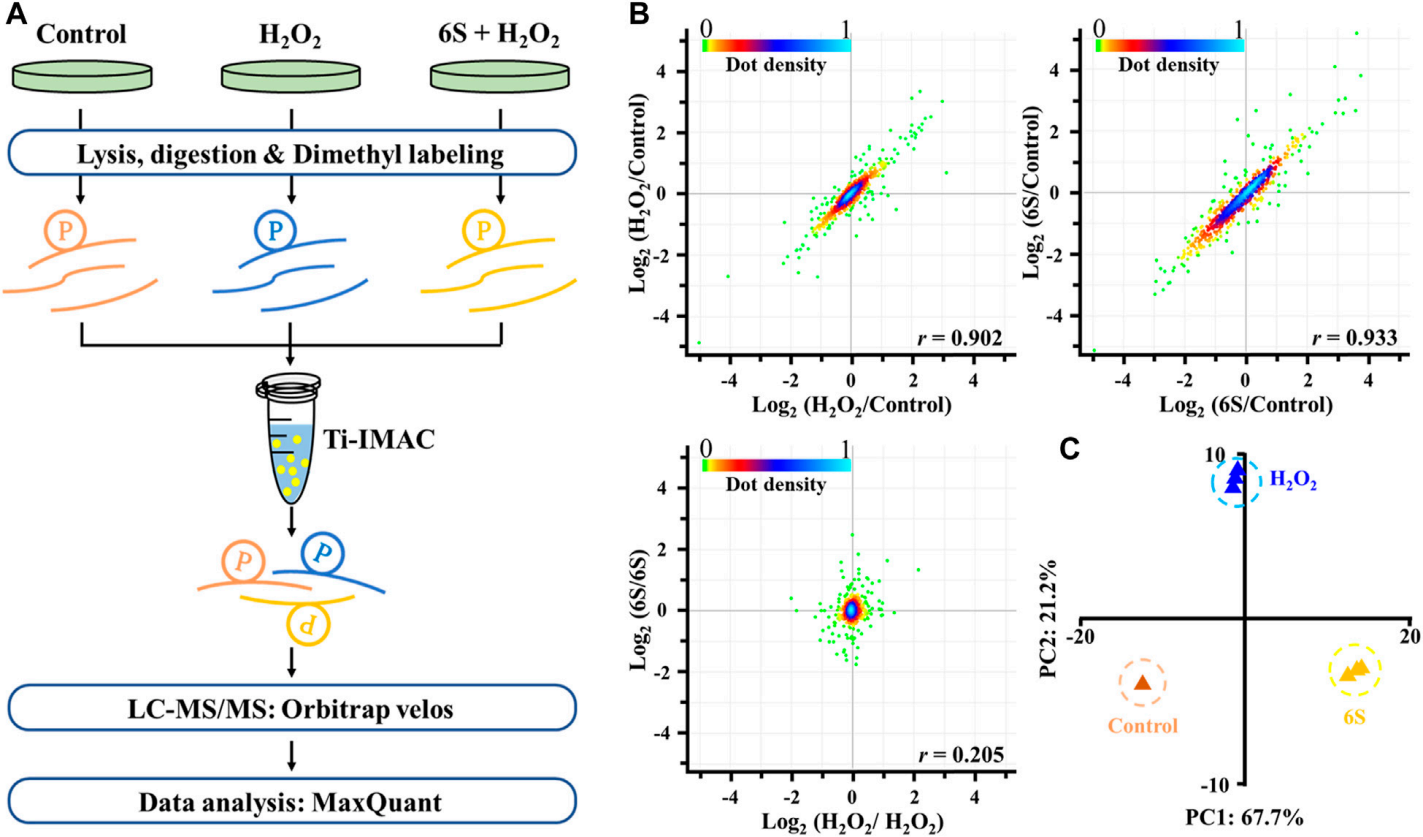
Mass spectrometry-based quantitative phosphoproteomic profiling of H_2_O_2_ injury and 6S protection. **(A)** Workflow of the mass spectrometry-based phosphoproteomic analysis. Digested peptides from three different experimental conditions were dimethyl-labeled, combined, and enriched for phosphopeptides using Ti-IMAC microspheres. Finally, the phosphopeptides were analyzed by LC-MS/MS. **(B)** Representative null comparisons (ratio of two replicates for H_2_O_2_ group and 6S group) displayed markedly distinct patterns from true comparisons (ratios of H_2_O_2_/control and 6S/control). **(C)** Principal-component analysis of all identified phosphorylation sites showed that samples cluster tightly according to biology. The log2 ratios of phosphorylation sites in the control group defaulted to 0.

To discover the action mechanism by which 6S protects VSMCs from oxidative stress-induced apoptosis, high-resolution MS-based phosphoproteomics in combination with quantification via stable isotope dimethyl labeling technology was applied to identify the global changes of protein phosphorylation in cells exposed to H_2_O_2_ with 6S pre-treatment or not (Figure 3A; three replicates). In total, 1,730 phosphorylation sites in 1,236 phosphopeptides were quantified (Supplementary Table S1, <1% false discovery rate (FDR)). The replicate data of the H_2_O_2_ group and 6S protection group were compared to differentiate experimental variances. The null comparisons of the biological replicates showed random distribution with a Pearson *r* of 0.205 (Figure 3B). In contrast, data comparisons of the H_2_O_2_ group and 6S protection group showed significant differences between the control group but consistency between replicates with Pearson *r* of 0.902 and 0.933, respectively (Figure 3B). Principal-component analysis (PCA) of all phosphorylation sites further verified the reproducibility of these results.

**FIGURE 4:**
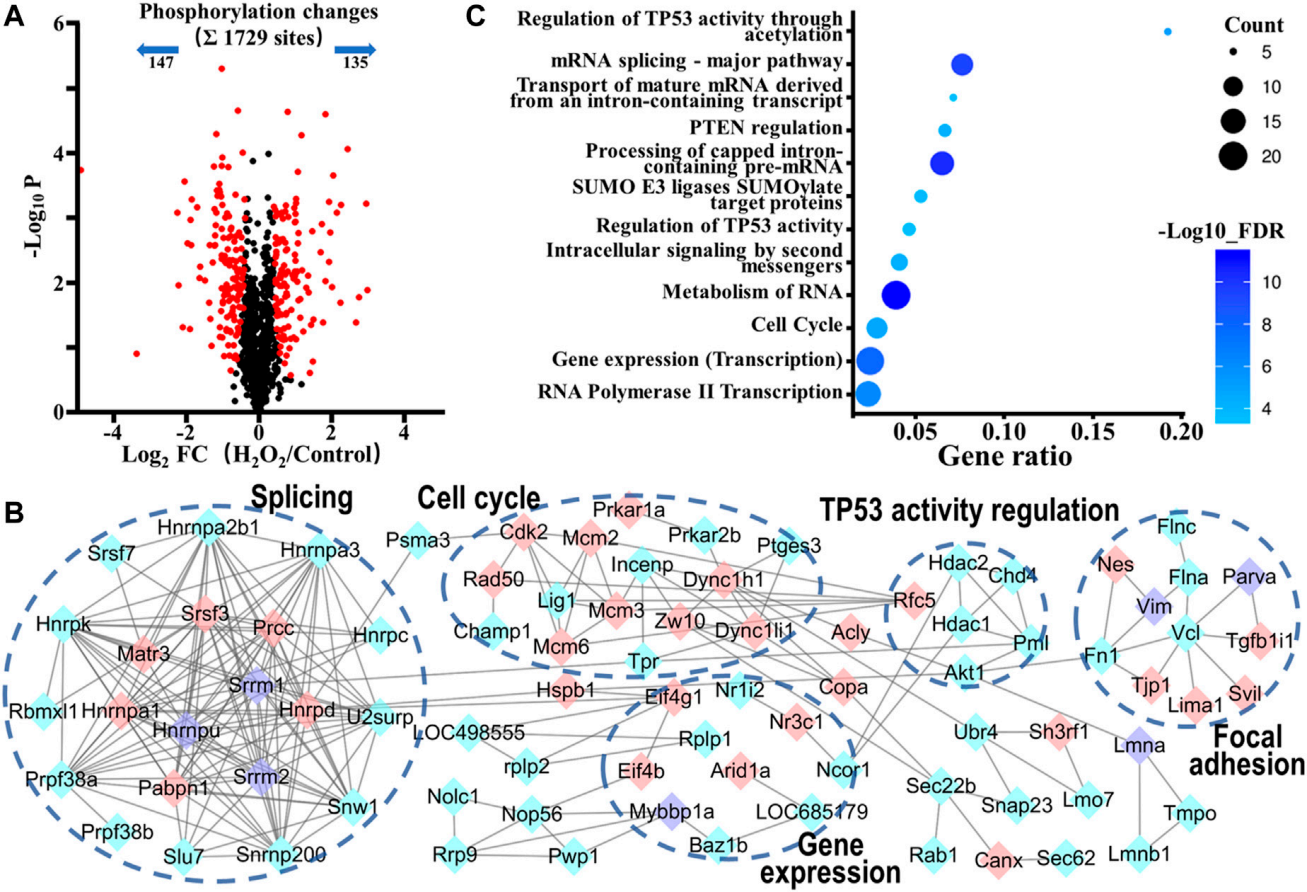
Phosphoproteomic analysis of the action mechanism of oxidative stress-induced apoptosis in VSMCs. **(A)** A volcano plot showed that 282 localized phosphorylation sites changed significantly (FDR < 0.05, S0 of 0.5) upon H_2_O_2_ treatment. **(B)** Protein-protein interaction map of phosphoproteins whose sites were significantly changed upon H_2_O_2_ treatment (STRING combined interaction score >0.7). Pink nodes represented their phosphorylation sites were upregulated, blue nodes represented their phosphorylation sites were downregulated, while purple nodes had both upregulated and downregulated phosphorylation sites. **(C)** The Gene Ontology- (GO-) based Reactome pathways enrichment of the altered phosphoproteins.

We next analyzed the phosphorylation changes upon H_2_O_2_ induced apoptosis. To assess statistical significance, an FDR-controlled t-test (FDR < 0.05, S0 of 0.5) was performed on localized sites (localization probability >0.7) that were observed in a minimum of two replicates. As a result, a total of 282 sites (16.3% of the 1729 sites) belonging to 175 phosphoproteins changed significantly upon oxidative stress (147 sites down and 135 sites up; Figure 4A and Supplementary Table S2). Global protein interaction analysis of changing phosphoproteins (STRING combined interaction score >0.7) in combination with Gene Ontology- (GO-) based Reactome pathways enrichment of the extracted network using the STRING database had revealed that oxidative stress damage in VSMCs was highly related to the processes of regulation of TP53 activity through acetylation (RNO-6804758, FDR = 6.8E-06), cell cycle (RNO-69278, FDR = 2.0E-05), gene expression (RNO-74160, FDR = 1.2E-08) and RNA-related processes such as RNA metabolism (RNO-8953854, FDR = 4.8E-12), mRNA Splicing (RNO-72163, FDR = 5.1E-11), and RNA polymerase II Transcription (RNO-73857, FDR = 6.8E-07) (Figures 4B,C). Tumor suppressor p53, a transcription factor expressed by the TP53 gene, is a key regulator in cell cycle arrest, apoptosis, senescence, or differentiation in response to various genotoxic and cellular stresses, including oxidative stress, by directly regulating the expression of hundreds of genes, products of which mediate various p53-dependent functions (Liu J. et al., 2015). Moreover, p53 is reported to be activated when interfering with the splicing machinery (Allende-Vega et al., 2013). Therefore, the results suggested a close connection of p53 and its related pathways with oxidative stress-induced VSMCs apoptosis.

As is well known, the regulation of p53 stability and activity is tightly based on posttranslational mechanisms. Without stresses, p53 is unstable and inactive because of the interaction with its negative regulators (such as MDM2 and MDMX), which suppress p53 activity and induce its proteasome-dependent degradation by ubiquitinating it. On the contrary, p53 is stabilized and activated in response to stresses because of the posttranslational modification changes on p53 or/and its negative regulators, which disrupt the interaction between them (Liu and Xu, 2011). We observed the downregulated phosphorylation of AKT1, HDAC1, HDAC2, and CHD4 upon H_2_O_2_ exposure. The inactivation of AKT1 is reported to obstruct phosphorylation of MDM2, which is essential for MDM2 to transfer into nucleolus and bind to p53, leading to the accumulation and activation of p53 (Rogulski et al., 2005). Acetylation could enhance the p53 stability. Histone deacetylase (HDACs) and CHD4 have been reported to act as important regulators of p53 deacetylation. Their inactivation leads to hyperacetylation of p53 and then boosts p53 stability and transcriptional activity (Ito et al., 2002; Polo et al., 2010; Chen et al., 2015). Besides, we also observed the upregulated phosphorylation of VIM, SH3RF1, ACLY, HNRPK, RAD50, and CDK, which were involved in p53 related cell cycle, apoptosis, and gene expression processes (Shin et al., 2001; Papagiannakopoulos et al., 2008; Wang et al., 2018). Those observations indicated that oxidative stress activated p53 and p53-mediated cell cycle arrest and apoptosis pathways.

**FIGURE 5:**
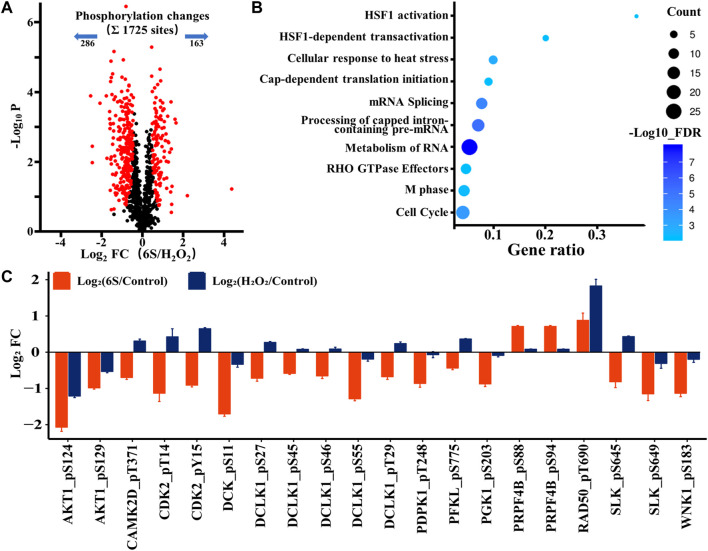
Phosphoproteomic analysis of the action mechanism of 6S protection in VSMCs. **(A)** A volcano plot showed that 449 localized phosphorylation sites changed significantly (FDR < 0.05, S0 of 0.5) with pre-treatment of 6S compared with the control group upon H_2_O_2_ treatment. **(B)** The Gene Ontology- (GO-) based Reactome pathways enrichment of the altered phosphoproteins. **(C)** Bar plots display the average log2 FC (FC: fold change) of quantified sites in the protein kinases that differently expressed with pre-treatment of 6S compared with the control group upon H_2_O_2_ treatment.

After demonstrating the possibility of p53 activation upon oxidative stress, we next investigated whether p53 participated in the protection process of 6S in VSMCs. Therefore, we looked at the phosphoproteomic difference occurring with 6S pre-treatment. A total of 449 sites (26.0% of the 1725 sites) belonging to 250 phosphoproteins displayed different changes upon oxidative stress (286 sites down and 163 sites up; Figure 5A and Supplementary Table S3), and 68 differently changed phosphoproteins (27.2%) were assigned to be involved in the process of response to stimulus (GO:0050896) by STRING database. Gene Ontology- (GO-) based Reactome pathways enrichment revealed that the protective effects of 6S were closely related to the biological processes of cell cycle (RNO-69278, FDR = 5.5E-04), RNA-related processes such as RNA metabolism (RNO-8953854, FDR = 1.1E-8), Processing of Capped Intron-Containing Pre-mRNA (RNO-72203, FDR = 6.2E-06), and mRNA Splicing (RNO-72163, FDR = 4.2E-5), and cellular stress response related processes such as cellular response to heat stress (RNO-3371556, FDR = 1.3E-03), HSF1-dependent transactivation (RNO-3371571, FDR = 5.9E-03), and HSF1 activation (RNO-3371511, FDR = 6.8E-03) (Figure 5B). As we all know, cell cycle and mRNA Splicing are highly related to p53. Moreover, several phosphorylation sites of the key proteins involved in HSF1 activation and HSF1-dependent transactivation, HSP90AA1, HSP90AB1, PTGES3, and CAMK2D, were observed as downregulated (Supplementary Table S3), implying the inactivation of heat shock factor 1 (HSF1), a transcription factor that was required for p53 nuclear importation and activation (Logan et al., 2009). Therefore, the activation of p53 might be inhibited by 6S pre-treatment.

As kinases are frequently involved in the oxidative stress and apoptosis processes and contain a high proportion of pharmacologically actionable targets, kinome perturbations are of special interest. The phosphorylation levels of 12 protein kinases were differently changed with pre-treatment of 6S compared to cells upon oxidative stress (Figure 5C). 6S showed a strong inhibition effect on the phosphorylation of AKT1, CAMK2D, CDK2, DCK, DCLK1, PDPK1, PFKL, PGK1, RAD50, SLK and WNK1, and whereas an activation effect on PRPF4B. Most of those kinases are related to apoptosis process and p53 activity. It is reported that RAD50 overexpression promotes cell death (Shin et al., 2001). CAMK2D (calcium/calmodulin-dependent protein kinase II- delta) and SLK (Ste20-like kinase) have been reported to play critical roles in the accumulation of p53 and induction of apoptosis (Cybulsky et al., 2009; Toko et al., 2010). The activation of CDK2 (Cyclin-dependent kinases2) and DCK (deoxycytidine kinase) during cell apoptosis can be regulated by p53 (Keszler et al., 2004; Roset and Gil-Gomez, 2009). The results suggested that 6S pre-treatment had inhibited the activation of p53 and its mediated apoptosis pathway induced by oxidative stress. This was further verified by western-blot experiments. H_2_O_2_ treatment significantly increased the expression levels of p53 and its downstream apoptosis-related proteins p21 (Gu et al., 2019), BAX and cleaved caspase 3 (p19), indicating the over-expression/activation of p53 and the serious VSMCs apoptosis. The expression levels were attenuated by pre-treatment with 6S (0.5, 2, and 8 μM) in a dose-dependent manner (Figure 2), bearing out that 6S protected VSMCs against oxidative damage by inhibiting p53-mediated cell cycle arrest and apoptosis.

### OXR1 Regulated p53 Expression *via* Opposite Regulation of SKP1

**FIGURE 6:**
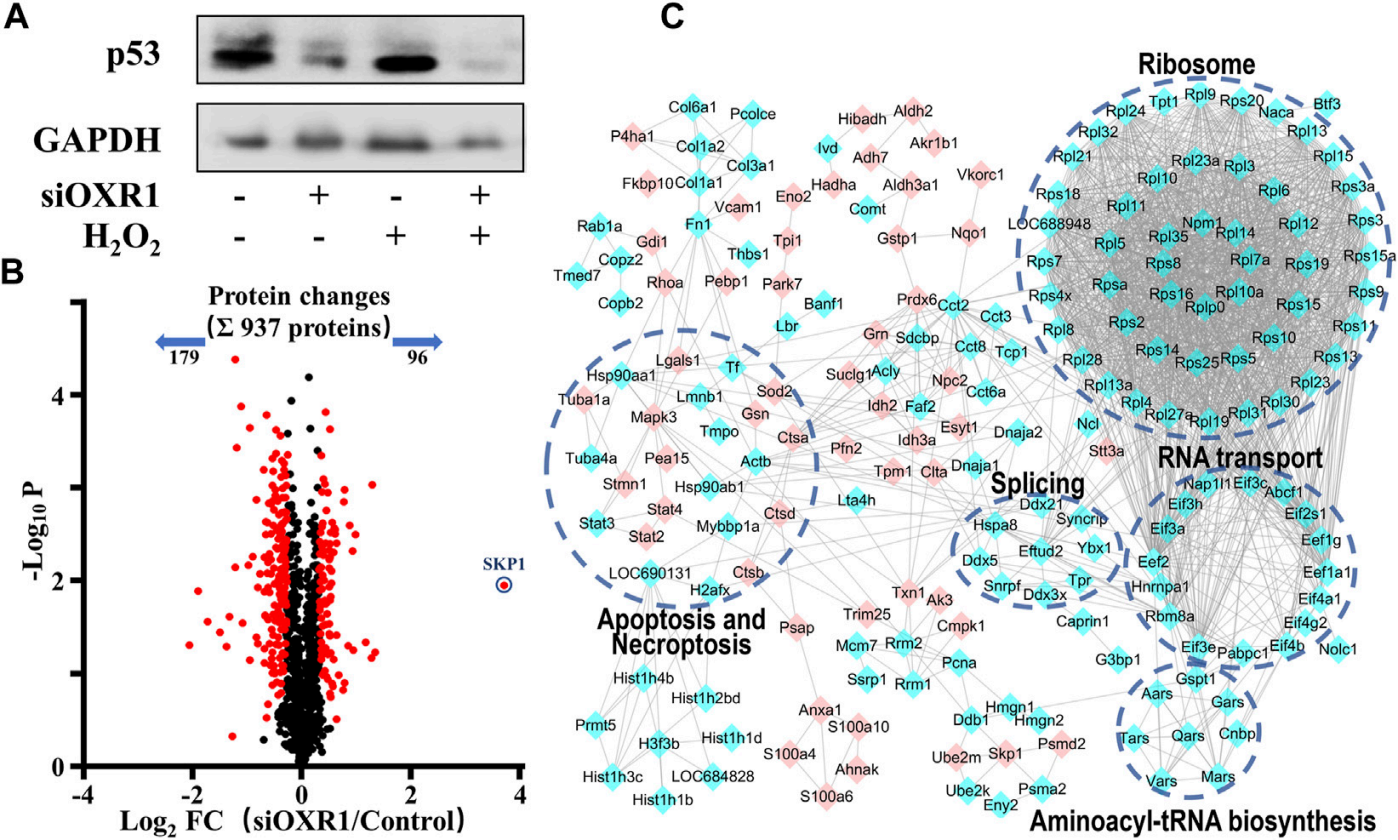
The regulation mechanism of p53 by OXR1. **(A)** The OXR1 can regulate the protein expression of p53. **(B)** A volcano plot showed that 275 proteins changed significantly (FDR <0.05, S0 of 0.5), among which SKP1 was observed to change most notably with a 12.9-fold upregulation. **(C)** The Gene Ontology- (GO-) based pathways enrichment of the altered proteins after OXR1 depletion.

The above results suggested a compact relationship between OXR1 and p53. We speculated that OXR1 could modulate cell cycle arrest and apoptosis in VSMCs by regulating p53 expression/activation, and this result was confirmed by siRNA experiments (Figure 6A; Supplementary Figure S2). A distinct decrease in p53 expression in both control and H_2_O_2_ damaged VSMCs after OXR1 silencing was obtained, whereas the p53 expression was significantly increased by H_2_O_2_ injury without OXR1 deprivation, suggesting that the expression of p53 protein was regulated by OXR1 in VSMCs. In this study, we found that 6S inhibited the upregulation of OXR1 upon H_2_O_2_ exposure (Figure 2), implying that oxidative stress upregulated OXR1 expression, which subsequently activated/upregulated p53, resulting in the cell apoptosis; 6S regulated p53 expression/activation and mediated VSMCs apoptosis though inhibiting OXR1 upregulation.

To further explore the mode of action by which OXR1 regulates p53 expression, the quantitative proteomic analysis was used to research the changes in the protein expression of VSMCs after OXR1 silencing. Using a threshold of FDR < 0.05 and S0 = 0.5, we found the expression levels of 96 and 179 proteins were increased and decreased by OXR1 depletion, respectively (Figure 6B, Supplementary Table S4). 5 GO terms were significantly associated with the OXR1 depletion (Figure 6C). We found the decreased levels of 48 ribosomal proteins and six translation initiation factors (eIFs), suggesting the inactivation of ribosome and RNA transport processes. We also found decreased levels of proteins involved in aminoacyl-tRNA biosynthesis and RNA splicing and differential expression levels of proteins related to apoptosis or necroptosis processes. Thus, our analysis indicated the crucial role of OXR1 in modulating gene expression, protein translation, and cell apoptosis. Additionally, OXR1 silencing resulted in a dramatic increase in the expression levels of SKP1 (S-phase kinase-associated protein 1, 12.9-fold), an essential component of the SCF (SKP1-CUL1-F-box protein) E3 ubiquitin ligase complex serving as an adapter that links the F-box protein to CUL1. The SCF complex mediates the ubiquitination of several proteins, and its functional specificity depends on the F-box protein acting as a substrate recognition component. It is well studied that the expression level of p53 was precisely regulated by multiple SCF E3 ubiquitin ligases (Johmura and Nakanishi, 2016; Cui et al., 2020) through promoting modification (ubiquitination or neddylation) and degradation of p53 (Abida et al., 2007; Sun et al., 2009; Verma et al., 2011). The result suggested that downregulating OXR1 expression induced SKP1 expression, which implied the enhanced assembly of SCF complex then resulted in the degradation of p53.

## Discussion

Apoptosis of vascular smooth muscle cells (VSMCs) significantly contributes to the pathogenesis of cardiovascular diseases, including inflammation, hypertension, atherosclerosis, and even myocardial infarction (Kumar et al., 2014; Mill et al., 2014). Recently, it has been shown that oxidative stress is one of the key factors that induce VSMCs apoptosis as ROS can act as second messengers in pathways leading to cellular apoptosis, DNA synthesis, and proto-oncogene mRNA expression in VSMCs (Irani, 2000; Cao et al., 2015), therefore, alleviating oxidative stress, and it induced VSMCs apoptosis is an effective measure to prevent cardiovascular disease.

6S is the major bioactive compound present in ginger and possesses serious pharmacological activities, such as neuroprotective, anti-oxidative, anti-inflammatory, anti-diabetic, anti-tumor, and anti-platelet aggregation (Dugasani et al., 2010; Sabina et al., 2010). 6S has been investigated to attenuate oxidative stress-induced apoptosis of neuronal and hepatic cells (Kim and Kwon, 2013; Kim et al., 2014) and prevent cardiovascular disease by protecting endothelial cells against oxidative stress-induced injuries (Wang et al., 2013). Our results demonstrated that 6S notably protected VSMCs from oxidative stress-induced apoptosis.

As one of the most important PTMs, phosphorylation plays crucial roles in almost all cellular processes, including the regulation of apoptosis. To discover the action mechanism by which 6S protects VSMCs from oxidative stress-induced apoptosis, mass spectrometry- (MS-) based quantitative phosphoproteomic analysis had demonstrated the crucial role of p53 in oxidative stress-induced apoptosis and the protective effect of 6S. It has been well established that overexpression of p53 leads to growth inhibition/apoptosis in VSMCs, while suppression of p53 activation abrogates the p53-induced apoptosis (Johnson et al., 1996). To date, the effects of 6S on p53 are limited to various tumors, in which 6S promotes tumor cells apoptosis through activating p53 and modulating downstream apoptotic signals (Kathiresan and Govindhan, 2016; Nazim and Park, 2019). Conversely, our results had demonstrated that 6S could effectively alleviate H_2_O_2_-induced VSMCs apoptosis by inhibiting the activation of p53 and p53-mediated cell cycle arrest and apoptosis pathways. However, the modulation mechanism of 6S on p53 expression/activation in VSMCs is still unclear.

OXR1 has always been considered a crucial participator for the early response to oxidative stress (Natoli et al., 2008) and an important antioxidant protein by regulating the expression of a variety of antioxidant enzymes (Li et al., 2014; Svistunova et al., 2019). A new feature of OXR1 as a regulator of p53 expression/activation was found in this research. In the state of oxidative stress, the OXR1 expression was upregulated in VSMCs, which subsequently activated/upregulated p53, resulting in cell apoptosis. Inhibition of the OXR1 upregulation prevented the overexpression/activation of p53. In this study, 6S exhibited effective inhibition on the upregulation of OXR1 induced by H_2_O_2_ in a dose-dependent manner, suggesting that 6S protected VSMCs apoptosis by inhibiting the overexpression of the OXR1-p53 axis.

At present, the regulation mechanism of p53 expression by OXR1 is completely unknown. In order to preliminarily find out the potential mechanisms, an MS-based quantitative proteomic analysis was applied to compare the protein expression changes after OXR1 silencing in VSMCs, and SKP1 was observed to change most notably with a 12.9-fold upregulation. SKP1 is an essential adaptor protein linking CUL1 and a series of F-box proteins to form the SCF complexes, which act as multi-protein E3 ubiquitin ligase complexes that promote ubiquitination and degradation of a large number of regulatory proteins involved in diverse processes (Liu Y. Q. et al., 2015). Accumulative evidence demonstrates that p53 is an important substrate of various SCF ubiquitin ligase complexes (Abida et al., 2007; Sun et al., 2009; Verma et al., 2011; Johmura and Nakanishi, 2016; Cui et al., 2020). When those SCF complexes are assembled and activated, p53 is recruited for ubiquitination or neddylation and subsequently degraded by the proteasome. The above results indicated that OXR1 depletion resulted in the overexpression of SKP1, which led to the enhanced assembly of SCF complexes and subsequently contributed to the inactivation and degradation of p53. Therefore, 6S protected VSMCs against oxidative stress-induced apoptosis via effectively inhibiting p53 activation, which might bound up with the downregulation of OXR1 and enhanced assembly of SCF complexes.

In summary, our results showed that 6S protected VSMCs against oxidative stress-induced apoptosis by regulating the OXR1-p53 axis. We had verified that the expression level of p53 was regulated by OXR1. In VSMCs, oxidative stress significantly promoted the expression of OXR1, resulting in the stabilization and activation of p53, which promoted VSMCs apoptosis. 6S could effectively prevent the upregulated expression of OXR1, which might lead to promoted ubiquitination or neddylation and subsequent degradation of p53 *via* enhanced assembly of SCF complexes, and, by extension, protect VSMCs against apoptosis. Therefore, 6S could be of therapeutic use in preventing VSMC damage-related cardiovascular diseases.

## Data Availability

The datasets presented in this study can be found in online repositories. The names of the repository/repositories and accession number(s) can be found below: http://proteomecentral.proteomexchange.org/cgi/GetDataset, PXD015127.
