# Corrigendum: 6-Shogaol inhibits oxidative stress-induced rat vascular smooth muscle cell apoptosis by regulating OXR1-p53 axis

**DOI:** 10.3389/fmolb.2023.1308875

**Published:** 2023-10-17

**Authors:** Jing Liu, Bin Li, Wenlian Li, Taowen Pan, Yunpeng Diao, Fangjun Wang

**Affiliations:** ^1^ College of Pharmacy, College of Integrative Medicine, Dalian Medical University, Dalian, China; ^2^ Key Laboratory of Separation Sciences for Analytical Chemistry, Dalian Institute of Chemical Physics, Chinese Academy of Sciences, Dalian, China; ^3^ Dalian Anti-Infective Traditional Chinese Medicine Development Engineering Technology Research Center, Dalian, China

**Keywords:** vascular smooth muscle cell, OXR1, p53, oxidative stress, apoptosis, 6-shogaol

In the published article, there was an error in Figure 6 as published. The protein bands of reference GAPDH was used incorrectly in Figure 6A. The corrected Figure 6 and its caption “FIGURE 6. The regulation mechanism of p53 by OXR1. **(A)** The OXR1 can regulate the protein expression of p53. **(B)** A volcano plot showed that 275 proteins changed significantly (FDR < 0.05, S0 of 0.5), among which SKP1 was observed to change most notably with a 12.9-fold upregulation. **(C)** The Gene Ontology- (GO-) based pathways enrichment of the altered proteins after OXR1 depletion” appear below.

**FIGURE 6:**
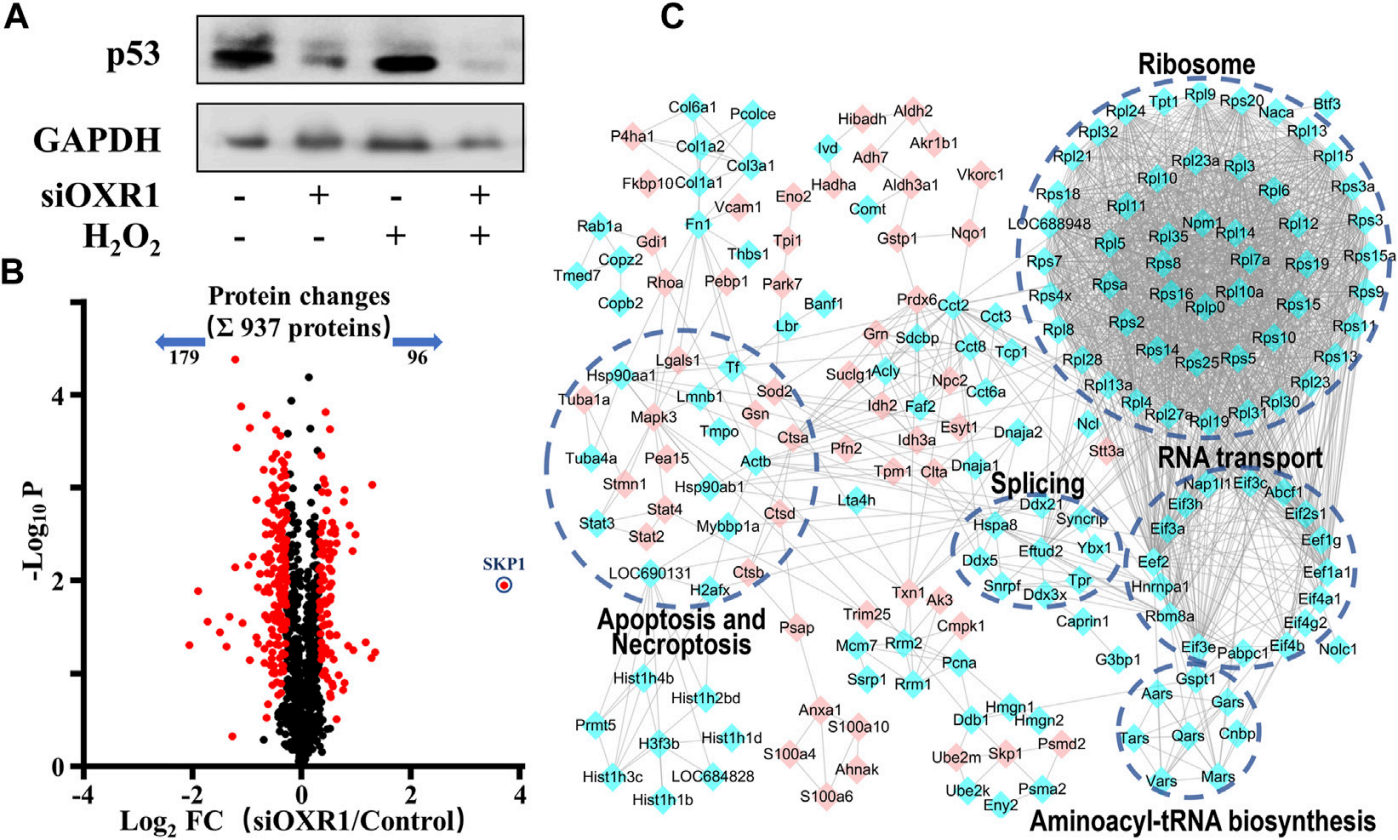
The regulation mechanism of p53 by OXR1. **(A)** The OXR1 can regulate the protein expression of p53. **(B)** A volcano plot showed that 275 proteins changed significantly (FDR < 0.05, S0 of 0.5), among which SKP1 was observed to change most notably with a 12.9-fold upregulation. **(C)** The Gene Ontology- (GO-) based pathways enrichment of the altered proteins after OXR1 depletion.

The authors apologize for this error and state that this does not change the scientific conclusions of the article in any way. The original article has been updated.

