# Supplementary material for: 6-Shogaol Inhibits Oxidative Stress-Induced Rat Vascular Smooth Muscle Cell Apoptosis by Regulating OXR1-p53 Axis
Source: Front Mol Biosci. 2022 Jan 31;9:808162. doi: 10.3389/fmolb.2022.808162 (PMC8841977; doi:10.3389/fmolb.2022.808162)
Supplement: Supplementary file 1 [file DataSheet1.docx]

**6-Shogaol** **Inhibits** **Oxidative Stress-induced Rat Vascular Smooth Muscle Cell Apoptosis by** **Regulating** **OXR1-p53 Axis**

**Jing Liu^1, 2, 3*#^, Bin Li^1, 3#^, Wenlian Li^1^, Taowen Pan^1, 3^, Yunpeng Diao^1,3^, Fangjun Wang^2, *^**

^1^ College of Pharmacy, College of Integrative Medicine, Dalian Medical University, Dalian, 116044, China.

^2^ Key Laboratory of Separation Sciences for Analytical Chemistry, Dalian Institute of Chemical Physics, Chinese Academy of Sciences, Dalian 116023, China.

^3^ Dalian Anti-Infective Traditional Chinese Medicine Development Engineering Technology Research Center, Dalian 116044, China.

^#^ Those two authors contributed equally to this work.

*** Correspondence**

Jing Liu, College of Pharmacy, Dalian Medical University, Dalian, 116044, China. E-mail: liujing8166@163.com

Fangjun Wang, Key Laboratory of Separation Sciences for Analytical Chemistry, Chinese Academy of Sciences (CAS), Dalian, 116023, China. E-mail: [wangfj@dicp.ac.cn](mailto:wangfj@dicp.ac.cn).

**Supplementary Figures**

**Fig. S1**


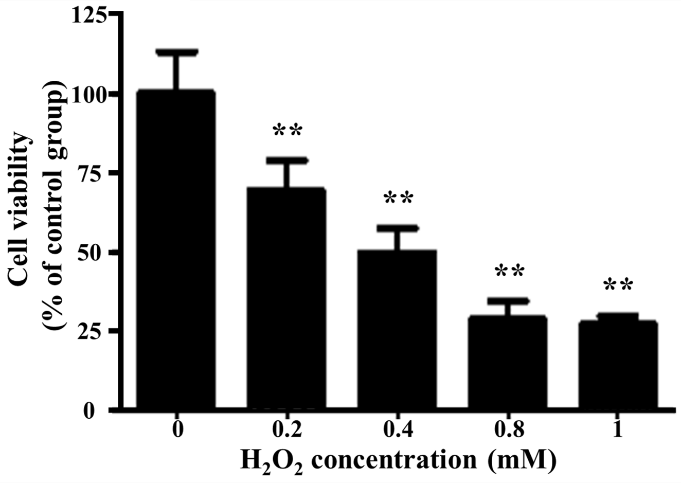


**Fig. S1** The effect of H_2_O_2_ on cell viability. VSMCs was incubated with 0, 0.2, 0.4, 0.8 or 1 mM H_2_O_2_ for 2h. All the cell viabilities were assessed using CCK-8 assay and expressed as mean ± S.D. (n = 6). **p < 0.01 versus control group.

**Fig. S2**

**
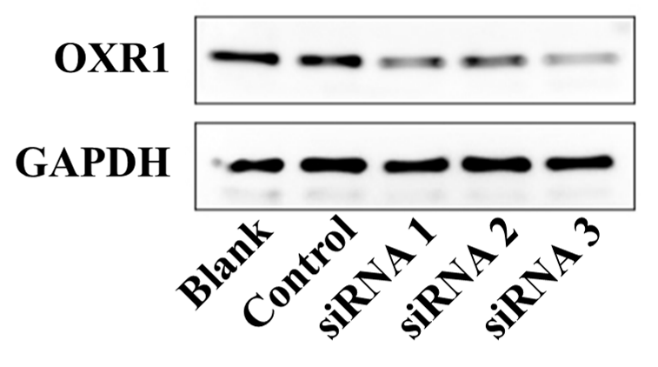
**

**Fig. S2** The expression level of OXR1 after siRNA transfection. 3 different OXR1 siRNA transfected VSMCs were obtained and the expression levels of OXR1 protein were measured. The siRNA3 transfected VSMCs was used in subsequent experiments.

**Supplementary Tables**

**Table S1** The quantification results of mass-spectrometry-based quantitative phosphoproteomic profiling of H_2_O_2_ injury and 6S protection. The VSMCs in control group were L labelled, the cells with H_2_O_2_ exposure were M labelled, and the cells with 6S pre-treatment before H_2_O_2_ exposure were H labelled.

**Table S2** The significantly changed phosphorylation sites during H_2_O_2_ exposure.

**Table S3** The significantly changed phosphorylation sites with 6S pre-treatment.

**Table S4** The significantly changed proteins after OXR1 depletion. The VSMCs in control group were L labelled, the cells with OXR1 depletion were H labelled.
